# Hyperemesis Gravidarum

**Published:** 1908-04-18

**Authors:** J. Halliday Croom

**Affiliations:** Professor of Midwifery, Edinburgh University.


### Hospital Clinics.

### HYPEREMESIS GRAVIDARUM.

By SIR J. HALLIDAY CROOM, M.D., F.R.C.P., F.R.C.S., Professor of Midwifery, Edinburgh University.

(A Clinical Lecture delivered at the Royal Maternity Hospital, Edinburgh.)

Gentlemen,—Within the last few weeks there have been several cases of well-marked hyperemesis gravidarum in this hospital, and as one has proved fatal I wish to draw your attention this afternoon to the whole condition.

Morning sickness, as you all know, is a very common concomitant of pregnancy in its earlier months. As a rule it begins after the first menstrual suppression, and continues more or less until the third month. It generally manifests itself in three varieties: (1) that in which there is vomiting and no nausea; (2) that in which there is nausea and no vomiting, and (3) where there are both. The condition is troublesome, but generally gives rise to no serious symptoms. Its treatment is various, and no one form presents any special value over another.

This afternoon I am not concerned with that form of vomiting. I wish to draw your attention to it when it has ceased to become a symptom, and has developed into a more or less grave disorder; where the vomiting has gone on to such an extent as to impair the patient's nutrition, and seriously compromise her life. Such a condition of matters is by no means infrequent, and our first object ought to be to distinguish the form of vomiting with which we have to deal. You saw some time ago a well-marked case of retroversion of the gravid uterus between the third and fourth month, associated with very well-marked vomiting which had lasted for six weeks. The patient was sent in solely on this account, because she could retain nothing, and was getting exhausted. And you will recollect that after replacing the gravid uterus the vomiting ceased, and she went out in a few weeks quite well.

Such a form of vomiting one would place under the heading of reflex; and this reflex variety is accounted for in various ways—not only by displacement, but in cases by over-distension of the uterus with hydramnios or twins; or, under some circumstances, with hydatidiform degeneration of the chorion—the so-called "hydatid mole." From want of any better classification such cases may be very conveniently ranged in the class of reflex. Whether the condition is produced by simple over-distension, or by some alteration in the metabolism, is a matter which at present seems to be somewhat shrouded in mystery. But, be the cause what it may, the fact remains that in such cases the removal of the apparent cause brings the hyperemesis to a conclusion. If in hydramnios with twins the uterus is emptied the symptoms cease, and in retroversion, if the uterus be replaced, in the majority of cases hyperemesis ends. Some have associated this condition with rigidity of the cervix and inflammation of the endometrium. I think in all probability these conditions are merely coincident; they may aggravate, but I do not think they cause the symptom which we are discussing.

The second variety of hyperemesis would fall, as Williams and Kaltenbach have both pointed out, under the heading of hysterical or neurotic. This class of cases is fairly easily recognised by the facts that there is no abnormality about the uterus or appendages; that the patient is probably of a neurotic or hysterical temperament; and that there is no abnormality in the urine, and no manifestation of any change in the liver. As a matter of fact, such cases usually get well, although I should like to mention to you a case in which this form of vomiting reduced the patient almost to the last extremity. Those who were attendant upon her as well as myself were very averse to interference, yet there was no alternative; with the emptying of the uterus the symptoms ceased. Such cases are extremely capricious. Assuming that they are hysterical in their origin, or at any rate due to increased nervous irritability, or diminished reflex control, they can ordinarily be managed by medicinal treatment and moral control, and they lend themselves to treatment by suggestion and the rest cure.

These two varieties to which I have referred seldom cause any very grave anxiety, because they can in the majority of cases, as I have already pointed out, be satisfactorily dealt with.

But the third variety—toxic vomiting—is quite another matter. And here I must refer you to the case that has been the text of this afternoon's lecture. The patient, a married woman, aged 33, primigravid, menstruated last at the end of May 1907. Two months after that she began to suffer from vomiting, which at first occurred only after meals, but after a few weeks became aggravated, and during October she vomited at short intervals during the day, irrespective of meals. In the beginning of November the vomiting and retching became almost constant, and she brought up mucus and blood. She came to this hospital when she was six months pregnant. On admission she was anæmic and emaciated, her lips were pallid, and the sclerotics somewhat jaundiced. There was no dropsy or cyanosis. The temperature was 96°, and the pulse 152 per minute, thin and very irregular. The stomach was not dilated, and there was no apparent abnormal condition of the alimentary tract. The liver dulness was normal. There was no erosion of the cervix nor any obvious pelvic abnormality to be made out on examination. Four ounces of urine were drawn off and examined carefully. It contained quantities of albumen, blood, and bile, while urates and granular blood casts were found in the deposit; there was no leucin or tyrosin. On the night of admission she was so weak and exhausted that one could not think of procuring abortion. It was decided to treat her with injections of saline solution and plenty of warm bottles. Afterwards she was given some bismuth and salol, followed by dilute milk, but she continued to vomit persistently. She was stimulated with strophanthus and strychnine, as the pulse indicated from time to time, but she died within twenty-four hours after admission.

The post-mortem examination showed the six months' pregnancy, with a small fibroid on the right lateral aspect of the uterus. The right kidney showed extensive fatty change, and the left kidney the same condition to some extent. The liver and spleen were normal, and so were the lungs. The stomach was small and contracted, and healthy in appearance. No trace of any food was found in any part of the alimentary canal. The walls of the venous plexus at the lower end of the œsophagus were thin and several branches had been ruptured, which would adequately account for the history of hæmatemesis during the last days before death. In this case there was nothing ante-mortem to suggest either a reflex or a neurotic cause, while the condition of the urine pointed to some toxic state. This was borne out at the autopsy, for the fibroid was too small to be a likely cause of reflex vomiting.

Severe vomiting is not infrequently associated during the later third of pregnancy with eclampsia, and at other times with acute yellow atrophy of the liver. It has, therefore, been thought that one toxin may be the common cause of these three conditions. This is to some extent supported by the fact that the lesions which take place in the kidneys and liver are somewhat similar in all three conditions. What this toxin is, and how exactly it acts, is the crux of the question. The whole subject is enveloped in doubt. There are, briefly, four sources from which the poison may be derived: the alimentary tract, the fœtus and placenta, the supposed ovarian secretion, and the liver. The alimentary tract may be the possible source in some cases, because often it contains matter which may give rise to toxins circulating in the blood; and certainly this, clinically, is to some extent supported by the benefit which often results from washing out the intestines. As in eclampsia, so in hyperemesis, the great bulk of evidence goes to show that the liver and kidneys are the sources of the poison. Post-mortems have abundantly shown, both in eclampsia and in hyperemesis, that extensive changes take place in the liver, often of a necrotic form.

Moreover, of recent years many cases of pernicious vomiting in pregnancy, in which minute and careful chemical observations have been made, point directly to the metabolism of the liver being at fault. It has been shown that while the total nitrogen output is unaltered, urea is diminished and ammonia increased. As you are aware, ammonia salts form the stage immediately preceding urea in the process of elimination of nitrogenous products. So that in this condition the functions of the liver may be at fault, rendering it unable to do its usual work of converting the nitrogenous material from the stage of ammonia to that of urea. On the other hand, we may suppose that the toxin is an acid, and that the increase of ammonia is an effort on the part of the body to protect itself against a condition of acid intoxication which would be incompatible with life.

With regard to treatment I have nothing especial to say in reference to the ordinary vomiting of pregnancy. That you can deal with as you please. It is often a troublesome and always a capricious symptom, and almost any of the usual forms of treatment can be adopted for it.

The reflex and neurotic forms are confined mainly to the early months, and when hyperemesis occurs in the later months it is most usually the toxic variety. Hyperemesis and eclampsia are frequently associated, and although emptying the uterus, in my opinion, is the most satisfactory method of dealing obstetrically with eclampsia and likewise with hyperemesis, yet this is not invariably the case, because in two patients who had eclamptic seizures, and in whom the uterus was emptied by *accouchement forcé*, although the eclampsia ceased, the hyperemesis persisted for a week.

It is apparent, therefore, that we may classify hyperemesis into the three varieties—namely, reflex, neurotic, and toxic; or, again, we may consider it as (1) that of the early months, and (2) that of the later months—the hyperemesis of the earlier months being most usually neurotic or reflex, and the hyperemesis of the later months being most usually toxic.

With regard to the treatment of the graver variety, hyperemesis gravidarum, with which we are at present concerned, I have little to say with regard to medicinal treatment. I have already referred to the reflex variety and pointed out that the removal of the cause is generally sufficient to cure the disease. In the neurotic form medicinal treatment should be directed to soothing the nervous system with various sedative remedies, and counteracting any condition of acidity by the administration of antacids and careful regulation of the bowels. Such cases often lend themselves well to suggestion and the rest cure, and, as Rosenthal has pointed out, isolation and intellectual control are of much value. On two occasions I can recollect that the mere suggestion of local interference to the patient has been sufficient to stop the symptom. But sometimes, even in the neurotic form, it proves so very uncontrollable and reduces the patient to such a degree of emaciation, that I have found, on one or two occasions, that the induction of abortion was the only remedy. In one case, however, where I induced abortion, the vomiting continued for at least a fortnight after the emptying of the uterus, and the patient remained for a long time in a very serious condition.

The treatment of the third or toxic variety is always to be regarded with considerable anxiety. When kidney involvement has been shown by careful examination of the urine, when there are indications of derangement of the liver, or both, and when there is increased temperature and an increased rapidity of the pulse, then the induction of abortion must be very early considered, and undertaken ere the patient reaches the condition of the case which has formed the basis of this lecture. And in the selection of your method of inducing abortion, all will depend whether you require to do so at once, or whether you have time. If the former, then the rapid method, by dilatation and evacuation at one sitting, will be necessary; whereas, if you have more time, the slower method can be adopted.

In any case, gentlemen, when you have indications of disease or derangement of the functions of the liver and kidneys, with a rise of pulse and temperature, be prepared always to interfere at once.

